# A theory of the brain: localist representation is used widely in the brain

**DOI:** 10.3389/fpsyg.2012.00551

**Published:** 2012-12-04

**Authors:** Asim Roy

**Affiliations:** Department of Information Systems, Arizona State UniversityTempe, AZ, USA

## On localist and distributed representations

In this article, I present the theory that localist representation is used widely in the brain starting from its earliest levels of processing. Page (2000) argued for localist representation and Bowers (2009) claimed that the brain uses grandmother cells to code for objects and concepts. However, neither Page (2000) nor Bowers (2009) claimed widespread use of localist representation in the brain. So this is a stronger position than that taken by either. To support the proposed theory, I present neurophysiological evidence, both old and new, and an analysis of localist and distributed representation definitions and models.

“Meaning and interpretation” on a stand-alone basis is the fundamental character of a localist unit. In arguing for the proposed theory, I bring to the forefront the “meaning and interpretation” aspect of localist cells and the evidence for it in the brain. I also show that localist and distributed models are not different structurally. In fact, any kind of model can be built with localist units. However, localist representation has no claim on the resulting properties of such models or what they can do.

### Definitions and what they mean

In cognitive science, distributed representation has the following property (Hinton et al., 1986; Plate, 2002):

- A concept is represented by a pattern of activity over a collection of neurons (i.e., more than one neuron is required to represent a concept.)
- Each neuron participates in the representation of more than one concept.

By contrast, in localist representation, each neuron represents a single concept on a stand-alone basis. The critical distinction is that localist units have “meaning and interpretation” whereas units in distributed representation don't. Many authors make a note of this distinction.

- Plate (2002): *“Another equivalent property is that in a distributed representation one cannot interpret the meaning of activity on a single neuron in isolation: the meaning of activity on any particular neuron is dependent on the activity in other neurons (Thorpe, 1995)*.”
- Thorpe (1995, p. 550): “*With a local representation, activity in individual units can be interpreted directly … with distributed coding individual units cannot be interpreted without knowing the state of other units in the network*.”
- Elman (1995, p. 210): “*These representations are distributed, which typically has the consequence that interpretable information cannot be obtained by examining activity of single hidden units*.”

Thus, the fundamental difference between localist and distributed representation is only in the interpretation and meaning of the units, nothing else. Therefore, any kind of model can be built with either type of representation.

### A classic localist model—is it structurally different from a distributed one?

**Figure 1:**
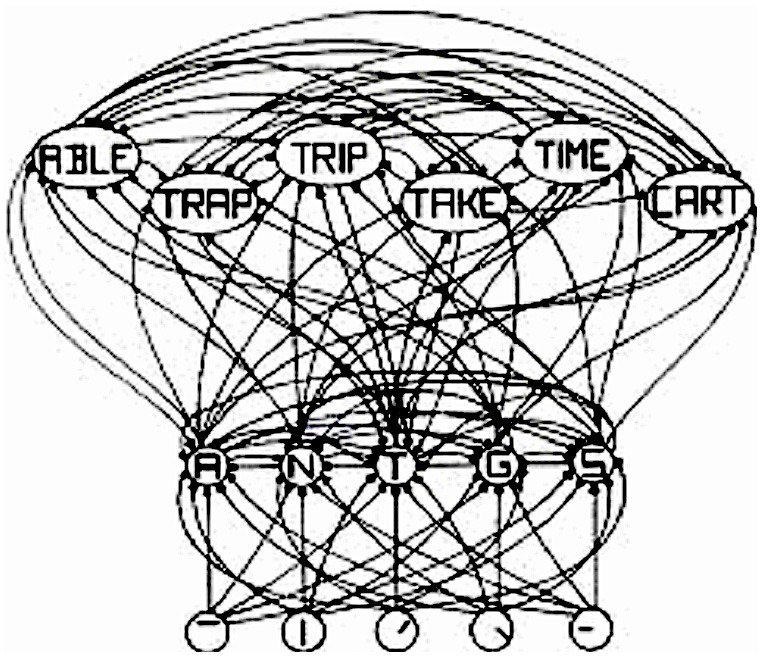
**Adapted from Figure 2 in “An Interactive Activation Model of Context Effects in Letter Perception: 1. An Account of Basic Findings,” by J. McClelland and D. Rumelhart, 1981, *Psychol. Rev.* 88, 380. Copyright 1981 by American Psychological Association.** Schematic diagram of a small subcomponent of the interactive activation model. Bottom layer codes are for letter features, second layer codes are for letters, and top layer codes are for complete words, *all in a localist manner*. Arrows depict excitatory connections between units; circles depict inhibitory connections.

The interactive activation (IA) model of McClelland and Rumelhart (1981), shown in Figure 1, is a classic localist model. The bottom layer has letter-feature units, the middle layer has letter units, and the top layer has word units. In the middle layer, the model has the same structure as a distributed model. That is, each word is represented by many letter units and each letter unit represents many different words. The same is true for the letter-feature layer. That is, each letter is represented by many letter-feature units and each letter-feature unit represents many different letters. So, regarding that defining property of distributed representation—where each entity is represented by many units, and each unit represents many different entities—a localist model is no different than a distributed one. That property is actually a property of the model, not of the units. The only difference between localist and distributed representation is whether individual units have “meaning and interpretation” or not. Here the IA model is a localist model simply because the letter-feature, letter, and word units have labels on them, which implies that they have “meaning and interpretation.”

### Can localist units respond to multiple concepts and still be localist?

A standard argument against localist representation (Plaut and McClelland, 2009; Quian Quiroga and Kreiman, 2010) is that for a cell to be localist, one has to show that it responds to one and only one stimulus class (e.g., one particular person or object). However, as the IA model shows, localist units can indeed respond to many different higher-level concepts. Thus, a letter unit will respond to many different words and a letter-feature unit will respond to many different letters and words. Thus, responding to many different concepts is not a property unique to distributed representation.

### Can there be redundant localist units?

An issue often raised in the context of grandmother cells is whether one and only one cell represents a concept or object (Gross, 1998). Note that grandmother cells are a special case of localist representation (Bowers, 2009). Localist representation has no claim that redundancy does not exist in the brain and Bowers (2009) also has no such claim regarding grandmother cells. The only test for a cell to be localist is that it has “meaning and interpretation” on a stand-alone basis.

## The evidence for localist cells in the brain—cells that have “meaning and interpretation”

### Cells in early processing stages have “meaning and interpretation” on a stand-alone basis

Research on a hierarchy of receptive fields is over four decades old and has produced Nobel Prize winners in medicine and physiology (Hubel and Wiesel, 1968). Receptive field neurons are found in all sensory systems—auditory, somatosensory, and visual. For example, they are found in all levels of the visual system—retinal ganglion, lateral geniculate nucleus, visual cortex, and extrastriate cortical cells. The major finding of this research is that receptive field functionality in all stages of processing can be interpreted. For example, in the primary visual cortex, there are simple and complex cells that are tuned to visual characteristics such as orientation, color, motion, and shape (Ringach, 2004). Here's a sampling of some recent findings on receptive fields.

#### Ganglion cells

Levick (1967) identified three types of ganglion cells in the rabbit retina: orientation selection, local-edge detection, and uniformity detection. Bloomfield (1994) also found orientation-selective amacrine and ganglion cells in the rabbit retina. Venkataramani and Taylor (2010) found more OFF-center orientation selective ganglion cells than ON-center ones in the visual streak of the retina.

#### Primary visual cortex

Usrey et al. (2003) found that 84% of the neurons in layer 4 of primary visual cortex in adult ferrets were orientation-selective simple cells with elongated receptive fields. Ringach et al. (2002) found contrast invariant edge kernels in both simple and complex cells in monkey primary visual cortex. Johnson et al. (2001, 2004, 2008) found that about 40% of all macaque V1 cells and 60% in layer 2/3 were color-selective. Martinez et al. (2005) found simple receptive fields exclusively in the thalamorecipient layers (4 and upper 6) in the cat's primary visual cortex and complex cells throughout the cortical depth. Gur et al. (2005) found a narrow band of direction- and orientation-selective cells located in the middle of layer 4C in V1 of alert monkeys showing use of very selective cells in early cortical processing.

Thus “meaning and interpretation” of cell activity exist starting at the lowest levels of sensory signal processing.

### Cells in later processing stages also have “meaning and interpretation” on a stand-alone basis

#### Hippocampal place cells

It's a tradition in neurophysiology to interpret the activity of cells in different brain regions. For example, there's four decades of research on hippocampal place cells that fire when an animal is in a specific location (O'Keefe and Dostrovsky, 1971; Moser et al., 2008). Recently Ekstrom et al. (2003) had epilepsy patients play a taxi driver computer game. They found cells in the hippocampus that responded to specific spatial locations, in the parahippocampal region that responded to views of specific landmarks (e.g., shops) and in the frontal and temporal lobes that responded to navigational goals.

#### Medial temporal lobe cells

Neuroscientists have discovered cells in the medial temporal lobe (MTL) region of the human brain that have highly selective response to complex stimuli. For example, some MTL neurons responded selectively to gender and facial expression (Fried et al., 1997) and to pictures of particular categories of objects, such as animals, faces, and houses (Kreiman et al., 2000). Thomas et al. (2000) found similar category encoding in the inferior temporal cortex. Quian Quiroga et al. (2008) found a neuron in the parahippocampal cortex that fired to pictures of Tower of Pisa and Eiffel Tower, but not to other landmarks. Quian Quiroga and Kreiman (2010) found a neuron firing to a spider and a snake, but not to other animals. Quian Quiroga et al. (2009) found a neuron in the entorhinal cortex that responded (p. 1308) “*selectively to pictures of Saddam Hussein as well as to the text ‘Saddam Hussein’ and his name pronounced by the computer …. There were no responses to other pictures, texts, or sounds*.” Koch (2011, p. 18, 19) reports finding similar MTL cells: “*One hippocampal neuron responded only to photos of actress Jennifer Aniston but not to pictures of other blonde women or actresses; moreover, the cell fired in response to seven very different pictures of Jennifer Aniston. We found cells that responded to images of Mother Teresa, to cute little animals and to the Pythagorean theorem*, a^2^ + b^2^ = c^2^.” Note that the “interpretation and meaning” of these cells did not depend on the activity of other cells. Quian Quiroga et al. (2008) estimate that 40% of MTL cells are tuned to such explicit representation.

#### The Cerf experiment

The experiment by Cerf et al. (2010) is quite revealing because it involves continuous interpretation of single cell activities. Here, epilepsy patients played a game to control the display of two superimposed images through four MTL neurons. Before the experiment, the researchers identified four MTL neurons in each patient that responded selectively to four different images. One of the four images was randomly selected to become the target image. Each trial started with a short display of the target image (say of Jennifer Aniston) followed by an overlaid hybrid image of the target and one of the other three images (a distractor image, say of James Brolin). The patient was then told to enhance the target image by focusing his/her thoughts on it. The initial visibility of both images was at 50% and the visibility of an image was increased or decreased every 100 ms based on the firing rates of the four MTL neurons. In general, if the firing rate of one neuron was higher compared to the other, the image associated with that neuron became more visible. The trial was terminated when either one of the two images was fully visible or after a fixed time limit. The subjects successfully reached the target, which means the target image was fully visible, in 596 out of 864 trials (69.0%; 202 failures and 66 timeouts).

Here's an interpretation of the experiment. Suppose A is the target image and B the distractor. Enhanced firing of the A cell is equivalent to the patient saying: “I am thinking about image A.” However, not a single word is spoken and the computer adjusting the images could still determine what the patient meant to say simply from the firing of the A cell. In other words, the firing of that A cell had “meaning and interpretation.”

Note also that if the target image was of Jennifer Aniston, the corresponding cell did not have any dependency of interpretation on any of the other three cells and those cells were not part of a distributed representation for the Jennifer Aniston concept. The other three monitored cells could have been for any of the other objects shown to the patient, such as a building or a snake, and that would not have changed the interpretation of the Jennifer Aniston cell. These cells, therefore, had “meaning and interpretation” on a stand-alone basis.

## Conclusion

The only requirement for a cell to be localist is that it have “meaning and interpretation” on a stand-alone basis and that its meaning does not depend on the activations of other cells. From the evidence so far from neurophysiology, it would be fair to conclude that use of localist representation is fairly widespread in the brain, starting from the lowest levels of processing. And the evidence for such a theory of the brain is substantial and convincing at this point and spans decades of work in neurophysiology.
